# Role of Endothelial Dysfunction in Cardiovascular Diseases: The Link Between Inflammation and Hydrogen Sulfide

**DOI:** 10.3389/fphar.2019.01568

**Published:** 2020-01-21

**Authors:** Hai-Jian Sun, Zhi-Yuan Wu, Xiao-Wei Nie, Jin-Song Bian

**Affiliations:** ^1^ Department of Pharmacology, Yong Loo Lin School of Medicine, National University of Singapore, Singapore, Singapore; ^2^ National University of Singapore (Suzhou) Research Institute, Suzhou, China

**Keywords:** endothelial cell, gasotransmitters, hydrogen sulfide, inflammation, cardiovascular disease

## Abstract

Endothelial cells are important constituents of blood vessels that play critical roles in cardiovascular homeostasis by regulating blood fluidity and fibrinolysis, vascular tone, angiogenesis, monocyte/leukocyte adhesion, and platelet aggregation. The normal vascular endothelium is taken as a gatekeeper of cardiovascular health, whereas abnormality of vascular endothelium is a major contributor to a plethora of cardiovascular ailments, such as atherosclerosis, aging, hypertension, obesity, and diabetes. Endothelial dysfunction is characterized by imbalanced vasodilation and vasoconstriction, elevated reactive oxygen species (ROS), and proinflammatory factors, as well as deficiency of nitric oxide (NO) bioavailability. The occurrence of endothelial dysfunction disrupts the endothelial barrier permeability that is a part of inflammatory response in the development of cardiovascular diseases. As such, abrogation of endothelial cell activation/inflammation is of clinical relevance. Recently, hydrogen sulfide (H_2_S), an entry as a gasotransmitter, exerts diverse biological effects through acting on various targeted signaling pathways. Within the cardiovascular system, the formation of H_2_S is detected in smooth muscle cells, vascular endothelial cells, and cardiomyocytes. Disrupted H_2_S bioavailability is postulated to be a new indicator for endothelial cell inflammation and its associated endothelial dysfunction. In this review, we will summarize recent advances about the roles of H_2_S in endothelial cell homeostasis, especially under pathological conditions, and discuss its putative therapeutic applications in endothelial inflammation-associated cardiovascular disorders.

## Introduction

Currently, cardiovascular disease is identified to be a major cause of people death around the world, and this situation is estimated to remain for many years to come, thus bringing a considerable burden to the world’s health resource (Mathers and Loncar, 2006). It is well known that poor diet, smoking, obesity, and physical inactivity are various modifiable risk factors for cardiovascular diseases, all of which lead to a proinflammatory state (Allende-Vigo, 2010). Actually, a wide range of evidence supports a crucial role of inflammatory response in the pathogenesis of cardiovascular diseases through driving endothelial cell activation/dysfunction (Carter, 2012). Therefore, it is not unexpected that huge efforts have been made to identify therapeutically potential targets to halt endothelial cell inflammation.

The blood vessels are composed of connective tissue, fibroblasts, endothelial cells, and vascular smooth muscle cells (VSMCs). On the innermost side of blood vessels, the normal endothelium is a semipermeable layer between blood stream and blood vessel wall. Due to its tight specialized cell-to-cell junctions, the endothelium forms a barrier that selectively limits the movement of macromolecules (Rahimi, 2017). The barrier is critically involved in vascular tone, fluid homeostasis, and host defense (Zhang et al., 2018b). Activated endothelial cells may release various cytokines, chemokines, and growth factors that promote the proliferation, migration, and permeability of endothelial cells (Park-Windhol and D’amore, 2016). The endothelial cells with inflammatory phenotype cause inflammation in the blood vessels, resulting in endothelial dysfunction and following progression of cardiovascular diseases (Sun et al., 2016). In accordance with this notion, endothelial cell inflammation is directly responsible for various cardiovascular diseases, such as hypertension, atherosclerosis, aging, stroke, heart disease, diabetes, obesity, venous thrombosis, and intimal hyperplasia (Sun et al., 2017; Castro-Ferreira et al., 2018; Haybar et al., 2019; Zhong et al., 2019).

In the endothelium, hydrogen sulfide (H_2_S), the third endogenous gaseous molecule after nitric oxide (NO) and carbon monoxide (CO), is synthesized and observed (Pan et al., 2017). Over the last decade, the roles of H_2_S in the pathogenesis of endothelial dysfunction have grown exponentially. As a result, the current understanding of H_2_S-mediated endothelial cell functions in both heath and disease continues to deepen. However, the potential molecular mechanisms that underlie H_2_S-mediated cardiovascular homeostasis, especially endothelial inflammation, are not comprehensively elucidated. The present review focuses on the current progress regarding the roles of H_2_S in endothelial inflammation-related cardiovascular disorders including hypertension, atherosclerosis and diabetes. Furthermore, we will discuss the possible challenges for developing H_2_S-derived therapeutics to treat endothelial dysfunction in cardiovascular disorders.

## Endothelial Dysfunction and Inflammation

**Figure 1:**
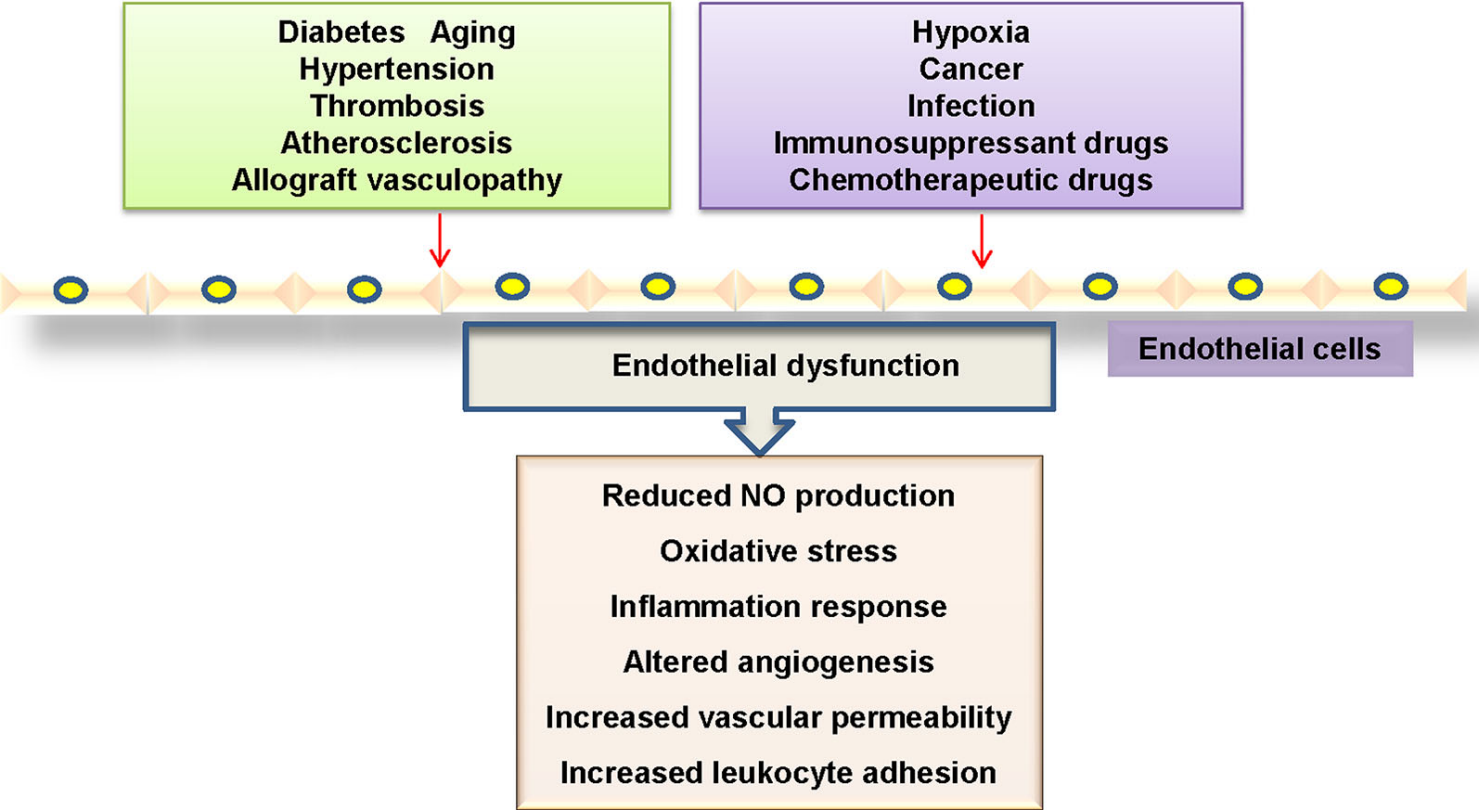
Mechanisms linked to endothelial dysfunction. Several key mechanisms that promote endothelial dysfunction.

The dysfunction of endothelial cells in the vasculature is profoundly implicated in the pathogenesis of cardiovascular disorders (Boulanger, 2016). Mounting evidence has shown that endothelial cell dysfunction is characterized by disrupted vascular tone and redox balance, and increased inflammatory reactions within the blood vessel wall (Ooi et al., 2018) (**Figure 1**). Most commonly, the impaired endothelium-dependent vasodilatation is defined as a hallmark of endothelial dysfunction, which is critically responsible for several cardiovascular disorders including diabetes mellitus, hypertension, atherosclerosis, aging and heart failure (Leung and Vanhoutte, 2017; Suryavanshi and Kulkarni, 2017). More recently, endothelial activation is also a prominent alteration in endothelial dysfunction, which refers to the upregulations of chemokines and adhesion molecules and other proteins involved in cell–cell interactions (Weber and Noels, 2011; Ng et al., 2018), thus leading to the prothrombotic and proinflammatory circumstance in the blood vessels.

In activated endothelial cells, the expressions of proinflammatory cytokines, chemokines, enzymes, and adhesion molecules are substantially upregulated (Baghai et al., 2018). It is highly possible that endothelial cell inflammation plays an important role in the pathogenesis of endothelial dysfunction in cardiovascular disorders. Therefore, identification of endothelial cell-derived inflammatory factors and its underlying mechanisms may be effective in preventing the progression of cardiovascular diseases.

## Regulation of Endothelial Function by H_2_S Under Physiological Condition

**Figure 2:**
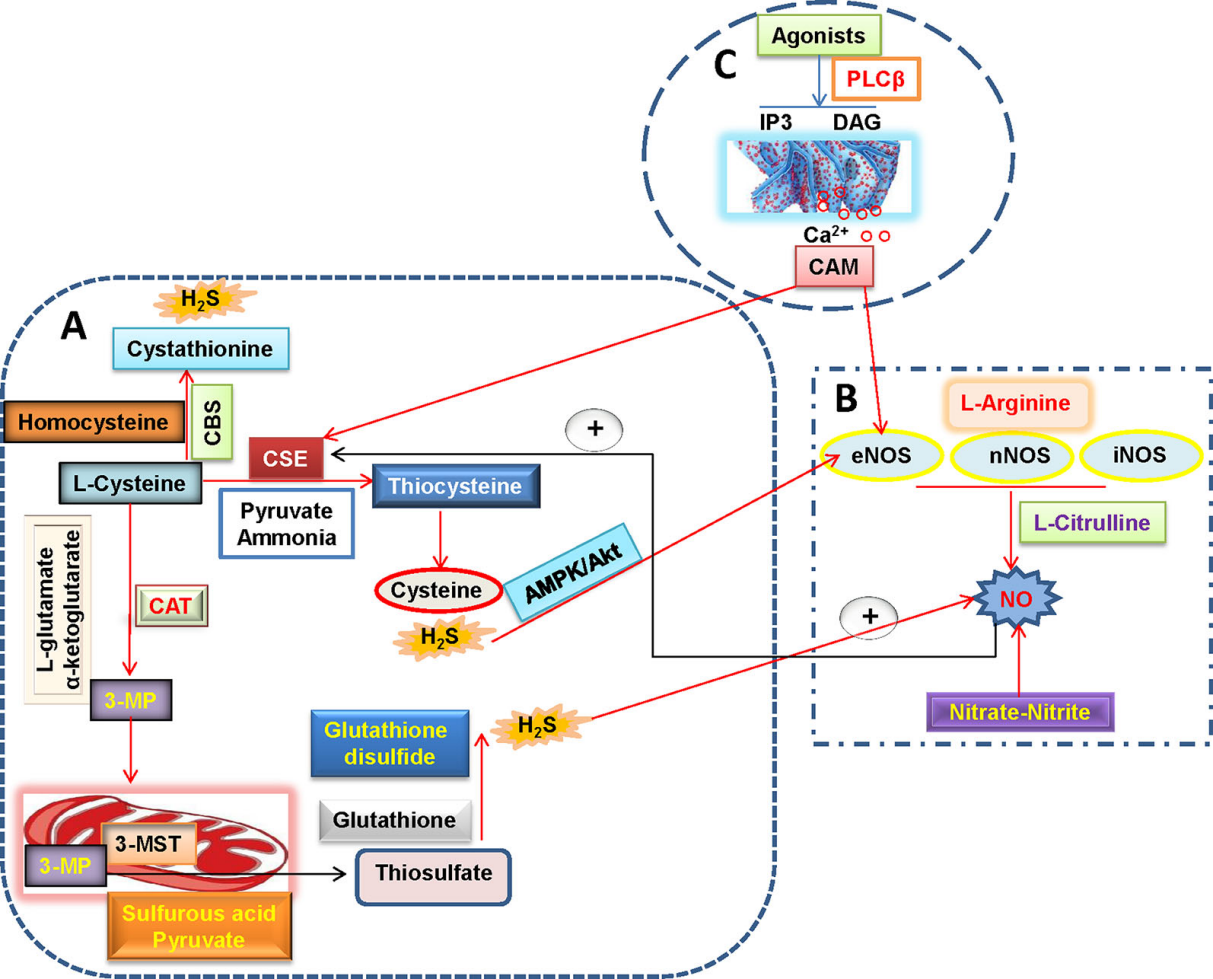
H_2_S and NO biosynthetic pathways in blood vessels. **(A)** L-cysteine is the substrate for the formation of H_2_S through three H_2_S-producing enzymes, L-cysteine is catalyzed by CSE to produce pyruvate, ammonia, and thiocysteine, the latter is then decomposed to cysteine and H_2_S. The endogenous H_2_S production by CBS is related with the condensation of homocysteine with L-cysteine, followed by the formation of cystathionine and H_2_S. Direct reaction of L-cysteine and α-ketoglutarate by CAT yields the release of 3-MP and L-glutamate, 3-Mercaptopyruvate is transported into the mitochondria where it is catalyzed to sulfurous acid, pyruvate and thiosulfate by 3-MST. In the presence of reduced glutathione, the thiosulfate is reduced to glutathione disulfide and H_2_S. It is well accepted that H_2_S can increase eNOS activity and thereby subsequent NO production directly or through AMPK/Akt signaling pathway. **(B)** NO is produced in all tissues by NOS-dependent (L-arginine-NO pathway) and -independent (nitrate-nitrite-NO pathway) pathways. A recently discovered pathway for NO generation is the serial reduction of the inorganic anions nitrate and nitrite. With the assistance of three isoforms of NOS including nNOS, eNOS, and iNOS, L-arginine is oxidized into L-citrulline with NO. NO is found ro increase CSE activity and expression and then stimulate H_2_S production. **(C)** In endothelial cells, vasoconstrictor agonists stimulate the release of Ca^2+^ and cause formation of calcium-calmodulin (CaM) *via* the PLCβ/IP3/DAG pathway. Then, CaM can simultaneously activate eNOS and CSE that yield NO and H_2_S, respectively. H_2_S, hydrogen sulfide; NO, nitric oxide; 3-MP, 3-mercaptopyruvate; CAT, cysteine aminotransferase; CSE, cystathionine γ-lyase; CBS, cystathionine β-synthase; 3-MST, 3-mercaptopyruvate sulfurtransferase; CaM, calcium-calmodulin; PLCβ, phospholipase Cβ; IP3, inositol-3-phosphate (IP3); DAG, diacylglycerol (DAG); eNOS, endothelial NO synthase; iNOS, inducible NO synthase; nNOS, neuronal NO synthase.

As the third endogenous gasotransmitter, H_2_S is primarily synthesized in mammalian tissues through enzymatic or non-enzymatic pathways (Li et al., 2011; Liu et al., 2012). The majority of endogenous H_2_S is produced by three enzymes including cystathionine γ-lyase (CSE), cystathionine β-synthase (CBS) and 3-mercaptopyruvate sulfurtransferase (3-MST) in mammalian tissues (Liu et al., 2011) (**Figure 2**). In the vascular endothelium, H_2_S is synthesized *via* the enzymatic metabolism of CBS/CSE using cysteine as the substrates (Tao et al., 2017; Mitidieri and Gurgone, 2019). Likewise, the involvement of 3-MST and cysteine aminotransferase (CAT) in endothelial generation of H_2_S has been demonstrated (Wang, 2012).

The regulation of vascular tone by H_2_S may be dependent on endothelium-independent and -dependent manners (Wang et al., 2015b). In the vasculature, H_2_S has been shown to induce vasodilation in aorta (Zhao et al., 2001), gastric artery (Kubo et al., 2007), mesenteric artery (Cheng et al., 2004), and internal mammary artery (Webb et al., 2008). The underlying mechanism by which H_2_S relaxes blood vessels is related with activation of vascular smooth muscle ATP-sensitive K^+^ (KATP) channels (Zhao et al., 2001), independently of the endothelium. The involvement of KATP channels in H_2_S-induced vasodilation is further confirmed by a finding that this relaxation is partially blocked by an inhibitor of KATP channels glibenclamide (Webb et al., 2008). Despite of these results, the exact mechanism of how KATP channels are directly activated by H_2_S still remains unknown. It is also reported that 4-aminopyridine-sensitive K^+^ channels are involved in H_2_S-induced relaxation in the rat coronary artery (Cheang et al., 2010). The H_2_S donor sodium hydrosulfide (NaHS) induces concentration-dependent vasorelaxation in both mesenteric arteries and aortas, which is blocked by the KCNQ-type Kv channel inhibitor XE991, suggesting the involvement of KCNQ channels in H_2_S-mediated peripheral artery relaxation (Schleifenbaum et al., 2010). Moreover, Ca^2+^ channels or sparks (Jackson-Weaver et al., 2015), Cl(-)/HCO(3)(-) channels (Kiss et al., 2008), the NO pathway (Ali et al., 2006), phospholipase A2 (D’emmanuele Di Villa Bianca et al., 2011), transient receptor potential (TRP) channels (White et al., 2013), and metabolic/mitochondrial effects (Kiss et al., 2008), are also suggested to be implicated in H_2_S-induced vasorelaxation. H_2_S appears to play an important role in vasorelaxation *via* multidimensional mechanisms. In the endothelium, recent studies have provided several lines of evidence to support that H_2_S might function as an endothelium-derived relaxing factor (EDRF), which shares many common traits with other EDRFs (Wang, 2009). Interestingly, the vasorelaxation actions of H_2_S are more remarkable in peripheral resistance arteries than in large-conduit arteries, the effects require the membrane hyperpolarization of both VSMCs and endothelial cells, as well as activation of endothelial intermediate conductance (IK(Ca)) and small conductance (SK(Ca)) potassium channels (Mustafa et al., 2011; Tang et al., 2013). The definition of H_2_S as an endogenous EDHF might shed light on possible therapeutic effects of H₂S on pathological abnormalities in the vascular system. Still, more extensive and mechanistic studies are needed to determine whether H_2_S is a new EDRF in the future.

**Figure 3:**
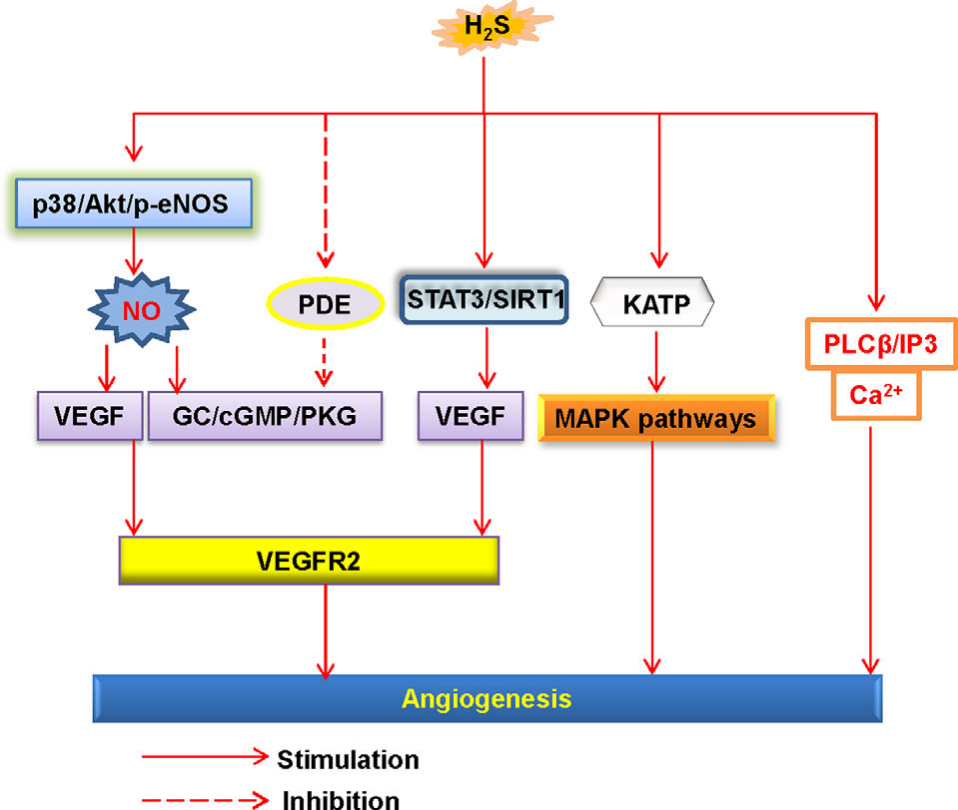
Schematic illustration of the underlying mechanisms of H_2_S-induced angiogenesis. H_2_S, hydrogen sulfide; NO, nitric oxide; Akt, protein kinase B; p38, p38 mitogen-activated protein kinases; eNOS, endothelial NO synthase; VEGF, vascular endothelial growth factor; VEGFR2, vascular endothelial growth factor receptor 2; PDE, phosphodiesterase; GC, guanylate cyclase; cGMP, cyclic guanosine monophosphate; PKG, protein kinase G; SIRT1, sirtuin 1; KATP, ATP-sensitive K+ channels; MAPK, mitogen-activated protein kinase; STAT3, signal transducer and activator of transcription 3; PLCβ, phospholipase Cβ; IP3, inositol-3-phosphate (IP3).

The endothelial cells also orchestrate tube formation and angiogenesis (Watson et al., 2017). H_2_S is reported to stimulate endothelial proliferation, migration, and angiogenesis (Wang et al., 2010b) (**Figure 3**). Furthermore, administration of H_2_S promotes angiogenesis in the Matrigel plug assay (Cai et al., 2007). However, it should be pointed out that high dose of H_2_S loses the ability to induce angiogenesis (Cai et al., 2007). In a rat model of chronic hindlimb ischaemia, intraperitoneal injection of the H_2_S donor NaHS at the lower dose significantly improves capillary density, angiographic scores, thus improving hindlimb blood flow (Wang et al., 2010a). In line with the results discussed earlier, higher dose of the H_2_S donor is found to be ineffective in this model (Wang et al., 2010a). On these grounds, we speculate that the effects of H_2_S donors in angiogenesis may range from physiological, cytoprotective effects (low concentration) to cytotoxic effects (which are generally apparent at higher concentrations) (Szabo and Papapetropoulos, 2011). From a genetic perspective, mutant mice lacking CSE exhibit a variety of pathological features, including delayed wound healing secondary to inhibition of angiogenesis (Papapetropoulos et al., 2009). It has been reviewed that several cellular signaling pathways, such as the PI3K/Akt pathway, the mitogen activated protein kinase (MAPK) pathway, and ATP-sensitive potassium channels, are involved in H_2_S-mediated angiogenic effects (Szabo and Papapetropoulos, 2011). In addition to this, further study has demonstrated that H_2_S specifically disrupts cys1045-cys1024 disulfide bond in vascular endothelial growth factor receptor 2 (VEGFR2) and then stimulates its conformation for angiogenesis (Tao et al., 2013). As a molecular switch, H_2_S is also reported to activate signal transducer and activator of transcription 3 (STAT3) (Kan et al., 2014), mammalian target of rapamycin (mTOR), and the VEGFR2 pathway (Zhou et al., 2016), then the endothelial cell proliferation and angiogenesis are observed. It is noteworthy that due to its proangiogenic effects, H_2_S might lead to pathological angiogenesis in atherosclerotic plaques, thus facilitating plaque vulnerability (Van Den Born et al., 2016). In spite of this, therapeutic angiogenesis is important for wound healing, organ ischaemia, or the reperfusion of previously ischaemic organs (Caporali and Emanueli, 2011; Dulmovits and Herman, 2012; Ng et al., 2018). For this reason, the reparative angiogenesis by H_2_S may provide novel therapeutic avenues for post-ischemic neovascularization. Due to the physiological importance of H_2_S in the endothelium, further research is indispensable to examine the novel roles of endogenous H_2_S in the regulation of cardiovascular functions.

## Role of H_2_S in Endothelial Inflammation

Using intravital microscopy, H_2_S donors are found to attenuate the leukocyte adherence in rat mesenteric arteries induced by aspirin, this effect may be likely dependent on activation of KATP channels (Zanardo et al., 2006; Zuidema and Korthuis, 2015). In accordance with this, blockade of endogenous H_2_S exacerbates leukocyte-mediated inflammation in the endothelium (Zanardo et al., 2006). By contrast, NaHS promotes leukocyte rolling and adherence in mesenteric venules of mice with cecal ligation and puncture (CLP)-induced sepsis (Zhang et al., 2007). These conflicting results imply that H_2_S acts as a pivotal regulator of leukocyte activation under different inflammatory states. However, in recent years, more studies support that H_2_S could inhibit the process of endothelial cell inflammation (Wen et al., 2018). For instance, specific endothelial deletion of CSE is associated with the development of endothelial inflammation and atherosclerosis, effects that are reversed on treatment with a polysulfide donor (Bibli et al., 2019). H_2_S treatment reduces the increases in inflammatory mediators such as vascular cell adhesionmolecule-1 (VCAM-1), intercellular adhesionmolecule-1 (ICAM-1) and monocyte chemoattractant protein-1 (MCP-1) in endothelial cell induced by tumour necrosis factor-α (TNF-α), and the underlying mechanism of this protective effect is primarily mediated by inhibition of soluble TNF-α shedding and its relevant MCP-1 release (Perna et al., 2013). Similarly, exogenous H_2_S attenuates Ang II-induced inflammation response *via* inhibition of the nuclear transcription factor-κB (NF-κB) signaling pathway in endothelial cells (Hu et al., 2016). Inhibition of the NF-κB pathway is also required for H_2_S to attenuate pulmonary endothelial cell inflammation and subsequent pulmonary hypertension (Feng et al., 2017b). Endogenous H_2_S could directly induce sirtuin1 (SIRT1) sulfhydration and stability, thus reducing aortic inflammation and atherosclerotic plaque formation (Du et al., 2019). Deficiency of CSE increases endogenous sulfur dioxide (SO_2_) level in endothelial cells, and blockade of endogenous SO_2_ aggravates CSE knockdown-induced NF-κB pathway and its downstream inflammatory factors release in endothelial cells (Zhang et al., 2018a), suggesting that the increased endogenous SO_2_ generation might act as a compensatory mechanism for the downregulated CSE/H_2_S pathway in endothelial inflammatory response (Zhang et al., 2018a). It is concluded that the anti-inflammatory effects of H_2_S donors show tremendous promise for the treatment of endothelial inflammation-related cardiovascular disorders.

In response to proinflammatory cytokines, the leukocyte or macrophages are activated and recruited to the endothelium, thus causing the development of endothelial dysfunction-related cardiovascular diseases (Fang et al., 2013). Fortunately, H_2_S is found to alleviate vascular inflammation through various signaling pathways, including inhibition of NF-κB and nucleotide-binding oligomerization domain, leucine rich repeat, and pyrin domain-containing protein 3 (NLRP3) inflammasome, activation of KATP channels and voltage- and calcium-gated potassium (BKCa) channels (Fiorucci et al., 2005; Zanardo et al., 2006; Zuidema et al., 2010; Altaany et al., 2014; Bourque et al., 2018; Li et al., 2019). These possible mechanisms of H_2_S may explain that H_2_S can diminish vascular inflammation and attenuate the vascular injury, suggesting that the anti-inflammation effect of H_2_S is a benefit for cardiovascular protection. Next, we will discuss the beneficial roles of H_2_S-mediated suppression of endothelial dysfunction in cardiovascular disorders including atherosclerosis, diabetic cardiovascular complications and hypertension.

## H_2_S-Related Endothelial Dysfunction in Atherosclerosis

Atherosclerosis, a chronic vascular disease of large and medium arteries, involves various risk factors including lipid deposition, hypertension, inflammatory factors, and hyperhomocysteinemia, which synergistically elicit endothelial dysfunction (Baszczuk et al., 2014). Biochemical effects of these factors on the endothelium could lead to endothelial cell damage and vascular remodeling (Baszczuk et al., 2014). This important event induces endothelial inflammation, macrophage differentiation, foam cell formation, platelet deposition, and thrombus formation (Chistiakov et al., 2017; Rahman and Woollard, 2017). As such, correction of endothelial dysfunction could be a therapeutic strategy for management of atherosclerosis.

In recent years, considerable evidence indicates that the downregulated CSE/H2S pathway plays a pathophysiologic role in the development of atherosclerosis (Kanagy et al., 2017; Fan et al., 2019). CSE-knockout mice fed with atherogenic diet exhibt more severe atherosclerosis, suggesting that the disturbed CSE/H2S pathway predisposes the animals to the development of atherosclerosis (Mani et al., 2013). Macrophage inflammation directly contributes to necrotic core formation and plaque instability in atherosclerosis (Kavurma et al., 2017). In oxidized low density lipoprotein (ox-LDL)-treated macrophage, the levels of CSE mRNA and protein expression, as well as H2S production are remarkably decreased, thus, this finding indicates that alterations of the CSE/H2S pathway plays an important role in ox-LDL-stimulated macrophage inflammation and atherosclerosis (Wang et al., 2013b). It is worth noting that CBS deficiency may cause hyperhomocysteinemia, which is an independent risk factor for the development of atherosclerosis (Zhang et al., 2012a; Yuan et al., 2017). In transgenic CBS-deficient mice, the emegence of hypercholesterolemia accelerates atherosclerotic lesions via oxidative stress and inflammatory monocyte generation (Zhang et al., 2009). On the other hand, vascular calcification and neointimal hyperplasia are also involved in the progression of atherosclerosis (Durham et al., 2018; Yu et al., 2018). Not surprisingly, the production of H2S and CSE protein expression are obviously decreased in rats with vascular calcification (Wu et al., 2006). The CSE expression and H2S production are impaired during the development of balloon injury-induced neointimal hyperplasia in rats, and this effect is obviously reversed by H2S treatment (Meng et al., 2007). In mice with high fat diet for 16 weeks, it is found that CSE protein level is downregulated in the liver, the lung, and the aortic endothelium, 3-MST was also reduced in the liver (Peh et al., 2014). By contrast, CBS expression was higher in the liver and the kidney (Peh et al., 2014). These results suggest that an abnormal H2S pathway may be an important factor for the pathophysiology of metabolic disorders and atherosclerosis.

The anti-atherosclerotic mechanisms of H_2_S have been gradually described, including anti-inflammatory response, anti-oxidative action, endothelial function preservation, inhibition of foam cell formation and regulation of ion channels (Altaany et al., 2014; Mani et al., 2014; Xu et al., 2014; Wang et al., 2017; Barton and Meyer, 2019). Reduced CSE expressions at both mRNA and protein levels are detected in ox-LDL-treated endothelial cells and in aortas from apolipoprotein E knockout (ApoE^-/-^) mice (Leucker et al., 2017). In this study, the authors demonstrated that increased histone deacetylase 6 (HDAC6) downregulated CSE and H_2_S production *via* posttranslational modifications, thus leading to endothelial cell dysfunction and the development of atherosclerosis (Leucker et al., 2017). In cultured vascular endothelial cells, the expressions of miR-455-3p, endothelial nitric oxide synthase (eNOS) protein and NO production are augmented by H_2_S. Besides, H_2_S levels and miR-455-3p expressions are also increased in human atherosclerosis plaque, suggesting that the miR-455-3p/eNOS/NO axis is required to H_2_S to circumvent the development of atherosclerosis (Li et al., 2017). Genetic deletion of CSE exaggerates atherosclerosis in ApoE^-/-^ mice, and treatment of CSE-knockout mice with H_2_S inhibits the development of atherosclerosis (Mani et al., 2013), pinpointing that endogenous H_2_S may be of benefit in the treatment of atherosclerosis. In addition, the augmented expressions of selectins (P-selectin and E-selectin) and cell adhesion molecules (ICAM-1 and VCAM-1) are observed in vascular endothelial cells from CSE knockout mice (Mani et al., 2013). GYY4137, a novel slow-releasing H_2_S compound, retards atherosclerotic plaque formation and partially restores endothelium-dependent relaxation in ApoE^-/-^ mice through decreasing vascular inflammation and oxidative stress (Liu et al., 2013). Preconditioning with NaHS also grants a protection in atherosclerosis, as manifested by decreased atherosclerotic plaque size and aortic ICAM-1 levels (Wang et al., 2009). Supplementation with H_2_S ameliorates, while inhibition of H_2_S formation intensified aortic CX3CR1 and CX3CL1 expressions and the formation of atherosclerosis (Zhang et al., 2012b). Recently, H_2_S induces S-sulfhydration of kelch-like ECH-associated protein 1 (Keap1) and nuclear factor erythroid 2-related factor 2 (Nrf2) dissociation from Keap1, followed by Nrf2 nuclear translocation and anti-oxidize effects in endothelial cells, contributing to the ameliorating effect of H_2_S on atherosclerosis in the context of diabetes (Xie et al., 2016). Furthermore, in a mouse model of disturbed flow-induced atherosclerosis, application of H_2_S donor NaHS considerably attenuates the severity of atherosclerosis via upregulating expressions of angiotensin converting enzyme 2 (ACE2), thus converting pro-atherosclerotic Ang II to anti-atherosclerotic angiotensin 1-7 (Ang-(1-7)) (Lin et al., 2017). At the cellular level, NaHS promotes the expression of ACE2 to exert anti-inflammatory properties in lipopolysaccharide (LPS)-stimulated endothelial cells, as pretreatment with a selective ACE2 inhibitor DX600 abrogates the anti-inflammatory effect of NaHS (Lin et al., 2017). The results showed that endogenous H_2_S system was involved in the development of atherosclerosis. Exogenous H_2_S could confer beneficial effects on the pathogenesis of atherosclerosis.

Also, H_2_S is involved in shear stress and blood viscosity. The occurrence of atherosclerosis may be initiated due to changed patterns of blood flow and ensuing shear stress (Dunn et al., 2015). It is well established that atherosclerotic plaque formation in the endothelium is site specific, and disturbed blood flow formed at the lesser curvature of the aortic arch and branch points promotes plaque formation, whereas steady laminar flow at the greater curvature is indicated to be atheroprotective (Heo et al., 2016). The branches and curvatures of the blood vessels are predisposed to endothelial dysfunction and atherosclerosis progression (Zhou et al., 2014). Under oscillatory shear stress, H_2_S treatment inhibits monocyte adhesion to endothelial cells *via* activating the NO-producing Akt/eNOS signaling pathway (Go et al., 2012). Conversely, H_2_S impairs shear stress-induced dilation of isolated mouse coronary arteries by inhibition of NO generation (Chai et al., 2015). It is likely that both H_2_S and NO are implicated in the shear stress-induced atherosclerosis. However, further investigation is required to help us obtain more novel insights into the underlying mechanisms.

## H_2_S-Related Endothelial Dysfunction in Diabetic Vascular Complications

Circulating levels of H_2_S are markedly reduced in diabetic animal models, such as diabetic rats (Jain et al., 2010; Suzuki et al., 2011), diabetic mice (Brancaleone et al., 2008), and also in diabetic patients (Jain et al., 2013; Suzuki et al., 2017). However, the mRNA level of CSE in the aortas of diabetic rats is not altered (Denizalti et al., 2011). Likewise, the expressions of CSE, CBS, and 3-MST are unaltered in either high glucose-treated endothelial cells or in the aortas of diabetic rats (Jain et al., 2010; Coletta et al., 2015). On the contrary, it has been demonstrated that both high glucose and palmitate inhibit CSE expression and H_2_S production in rat aortic endothelial cells, while exogenous H_2_S could protect endothelial cells against apoptosis under high glucose and palmitate stimulation *via* suppressing oxidative stress, decreasing mitochondrial fragments and promoting mitophagy (Liu et al., 2017). The CSE expression and H_2_S content are significantly reduced in granulation tissues of wounds in obese diabetic mice when compared with control mice (Zhao et al., 2017). The expression of CSE and H_2_S level are reduced after renal ischemia/reperfusion injury in diabetes mellitus (Chen et al., 2018). In comparison with control mice, the H_2_S content and CSE expression in heart tissues of diabetic rats are also markedly lower (Guo et al., 2017). In progressive diabetic nephropathy, CSE expression is markedly reduced, whereas CBS expression is unaffected (Yamamoto et al., 2013). By contrast, the protein and mRNA expression of CBS are specifically decreased in the kidney, while CSE expression remains unchanged in obese diabetic mice (Liu et al., 2018). Interestingly, CSE expression is upregulated in cerebral microvessels of type I diabetic rats (Streeter et al., 2013). Although the data are conflicting, they raise the possibility that H_2_S may be a double-edged sword under diabetic pathophysiology. Certainly, more research is needed to determine the molecular mechanisms underlying the changed or unchanged expressions of H_2_S-generating enzymes/H_2_S under diabetic conditions.

Despite of the aforementioned results, recent study has demonstrated that 3-MST activity is inhibited in endothelial cells during hyperglycemia, leading to reduced H_2_S level, impaired angiogenesis, and suppressed mitochondrial function (Coletta et al., 2015). It is highly probable that inactivation of 3-MST and elevated H_2_S depletion are putative mechanisms for the decreased circulating H_2_S levels in hyperglycemic endothelial cells. The high glucose-incubated vascular rings exhibit impaired endothelium-dependent relaxation, and this effect is rescued by CSE overexpression or H_2_S supplementation (Suzuki et al., 2011). In the same study, they have also shown that the vascular rings from mice with gene knockout of CSE display an aggravated impairment of endothelium-dependent relaxation in response to hyperglycemia (Suzuki et al., 2011). It is anticipated that genetic modulations of CSE, CBS or 3-MST levels are effective approaches to experimentally investigate the roles of H_2_S in diabetic vascular complications.

Exposure to high glucose results in elevated ROS production and apoptosis, as well as decreased superoxide dismutase activity in endothelial cells, and all the above responses could be eliminated by pretreatment with H_2_S (Guan et al., 2012). Exogenous H_2_S alleviated the ROS overproduction and apoptosis in hyperglycemic endothelial cells through inhibiting necroptosis (Lin et al., 2018). In the aortas of diabetic rats, the connexin (Cx) 43 and 40 expressions are downregulated, while protein kinase C (PKC) and nicotinamide adenine dinucleotide phosphate-oxidase (NADPH) oxidase subunits are upregulated, H_2_S appears to be effective in attenuating these abnormalities (Zheng et al., 2010). The novel mitochondria-targeted H_2_S donors AP123 and AP39 are proven to prevent hyperglycemia-triggered oxidative stress and metabolic abnormalities in microvascular endothelial cells, suggesting that these compounds could be useful for the treatment of diabetic vascular complications (Gero et al., 2016). Induction of H_2_S by Ginkgolide B alleviates endothelial dysfunction *via* inhibiting oxidative stress and increasing NO bioavailability in diabetic rats (Wang et al., 2015a). It is likely that the cardiovascular protective effects of H_2_S in diabetes may be mediated by inhibition of oxidative stress.

Inhibition of the leptin/leptin receptor signal pathway contributes to the protective effects of H_2_S on high-glucose-induced injuries in endothelial cells (Wu et al., 2016). Pretreatment with H_2_S prevents high glucose-induced ICAM-1 levels as well as NF-κB activation in endothelial cells (Guan et al., 2013). Besides, stimulation of endothelial cells with high glucose significantly promotes the secretion of endothelin-1 with the concomitant suppression of H_2_S production, and administration of H_2_S attenuates the release of endothelin-1 induced by high glucose (Guan et al., 2015). The increasing recognitions of protective effects of H_2_S in high glucose-induced endothelial inflammation provide a new avenue of antagonism towards diabetic vascular complications.

In addition, high glucose/palmitate-induced excessive autophagy in endothelial cells is rectified by H_2_S, this may be mediated by the Nrf2-ROS-adenosine 5’-monophosphate (AMP)-activated protein kinase (AMPK) signaling pathway (Liu et al., 2016). However, another group demonstrates that exogenous H_2_S inhibits mitochondrial apoptosis and promotes mitochondrial autophagy, thus protecting endothelial cells against apoptosis induced by high glucose and palmitate (Liu et al., 2017). These contradictory results suggest that additional research is necessary to ascertain the role of autophagy in H_2_S-mediated protective actions on diabetic endothelial dysfunction.

## H_2_S-Related Endothelial Dysfunction in Hypertension

The abnormal levels of H_2_S have been found to be correlated with hypertension (Szabo, 2007; Whiteman and Winyard, 2011). Specifically, in a clinical study, patients with severe hypertension exhibited lower plasma H2S level (Meng et al., 2015). In subjects with pulmonary hypertension, both CSE expression and H2S level are significantly lower than those in healthy population (Sun et al., 2014). *In situ* hybridization analysis has shown that the expression of CSE mRNA is downregulated in the pulmonary arteries of rats with pulmonary hypertension (Xiaohui et al., 2005). Likewise, the reduced protein contents of CSE and CBS are detected in pulmonary artery endothelial cells from tobacco smoke-induced emphysema and pulmonary hypertension (Han et al., 2011). It has been revealed a marked reduction in CBS and CSE expression as well as H2S production in mesenteric artery and carotid artery from dexamethasone-induced hypertensive rats (D’emmanuele Di Villa Bianca et al., 2015). By contrast, the suppressed CBS expression and reduced H2S concentration in the kidney are observed in high salt-induced hypertension in Dahl rats (Huang et al., 2015). A decreased CSE/H2S activity is a potential contributor to the pathogenesis of maternal hypertension in preeclampsia (Wang et al., 2013a). Also, the plasma H2S level and CSE protein expression in thoracic aorta are all suppressed in spontaneously hypertensive rats (SHR) in comparison with normotensive rats (Yan et al., 2004; Ahmad et al., 2014). An intriguing study has illustrated that the blood pressure is enhanced by treatment with the combination of CSE inhibitor DL‐propargylglycine (PAG) or the CBS inhibitor aminooxyacetic acid (AOA) in rats, while either compound alone has no any effect on the arterial pressure, suggesting that H2S plays a critical role in regulating blood pressure (Roy et al., 2012). Despite that the expression of 3-MST is still uncertain under hypensive condition, 3-MST gene therapy improves renovascular dysfunction in response to hyperhomocysteinemia (Sen et al., 2012). Thus, a better understanding of the biochemical functions of the H2S-producing enzyme 3-MST as well as its roles in hypertension may lead to new therapeutic targets based on modulation of H2S production. Overall, these studies suggest that endogenous H2S dysregulation plays an important role in regulating hypertneison-associated pathological processes.

As mentioned above, a close relationship between H_2_S-related endothelial dysfunction and hypertension is confirmed by an observation that genetic deletion of CSE causes the development of hypertension in mice (Yang et al., 2008). In these CSE knockout mice, the endothelium-dependent relaxation of resistance mesenteric arteries is particularly impaired (Yang et al., 2008). In a mouse model of Ang II-induced hypertension, both aortic endothelial function and NO bioavailability are significantly attenuated, and these are reversed by treatment with H_2_S (Al-Magableh et al., 2015). Conversely, blockade of endogenous H_2_S exacerbates these abnormalities (Al-Magableh et al., 2015). In other studies, application of H_2_S donors decrease blood pressure, reverse vascular remodeling *via* suppressing VSMC proliferation, and collagen deposition in the blood vessels (Zhao et al., 2001; Li et al., 2008; Wang, 2012; Meng et al., 2015; Tomasova et al., 2015). H_2_S therapy markedly restores eNOS function and NO bioavailability in Nω-nitro-l-arginine methyl ester (L-NAME)-induced hypertensive rats (Ji et al., 2014). In agreement with this, the improvement of endothelial function by H_2_S is largely attributed to inhibition of oxidative stress, suppression of renin angiotensin system (RAS), downregulation of BMP4/COX-2 pathway, or activation of the PPARδ/PI3K/Akt/AMPK/eNOS pathway, thus contributing to the antihypertensive mechanism of H_2_S in renovascular hypertensive rats (Xue et al., 2015; Xiao and Dong, 2016; Xiao et al., 2018). In SHR, exogenous H_2_S administration significantly reduces blood pressure and abrogated damaged endothelial dysfunction *via* inactivation of NLRP3 inflammasome and oxidative stress (Li et al., 2019). H_2_S treatment blunts increases in systolic blood pressure and ameliorates endothelial dysfunction by inhibiting oxidative stress in lead-induced hypertensive rats (Possomato-Vieira and Goncalves-Rizzi, 2018). These results demonstrate that the H_2_S pathway may provide potential therapeutic target for treating different hypertension models.

**Figure 4:**
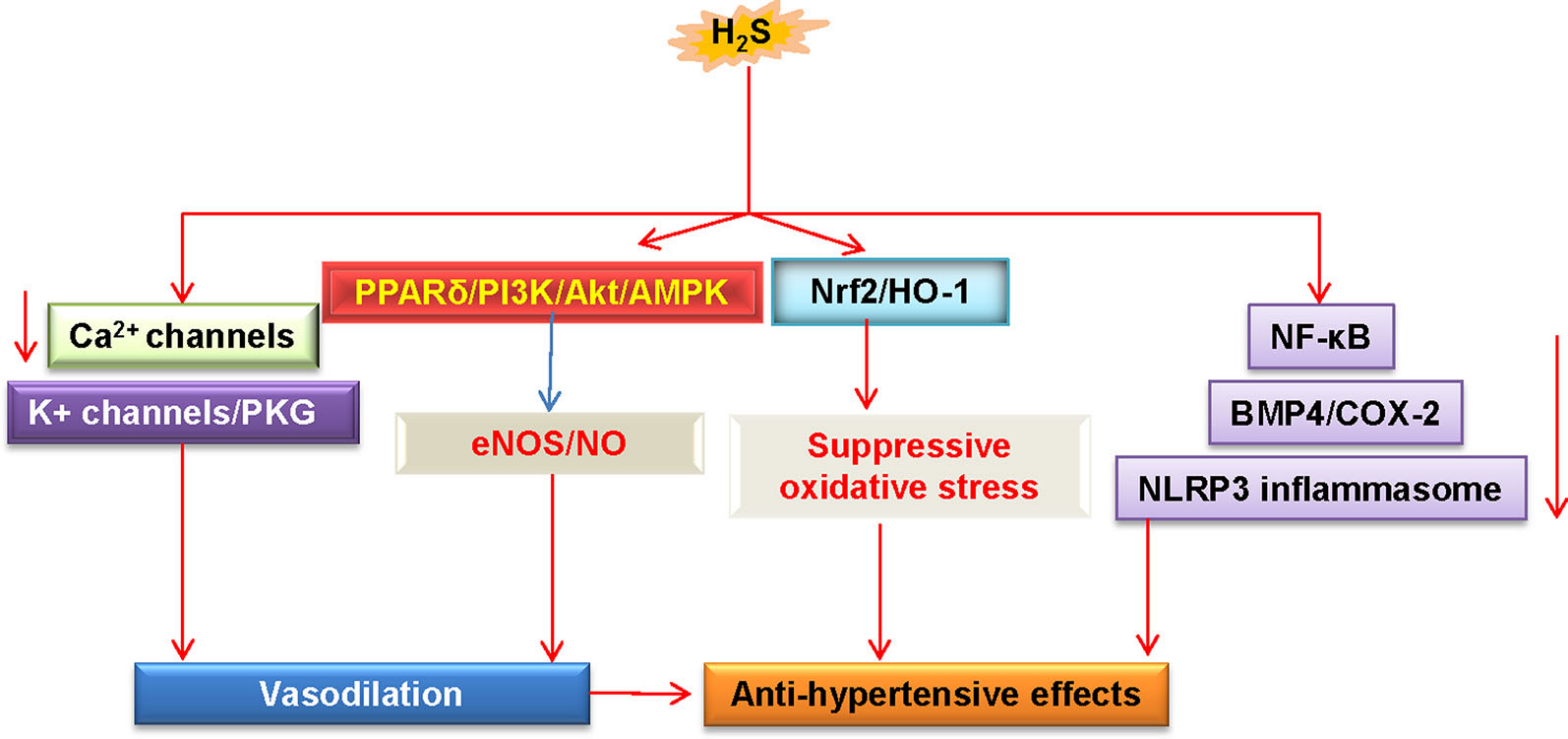
Schematic illustration of underlying mechanisms in which H_2_S protects against hypertension. H_2_S lowers high blood pressure *via* vasodilatation by activation of vascular KATP channels and inhibition of Ca^2+^ influx. The PPARδ/PI3K/Akt/AMPK signaling pathway participates in H_2_S-induced NO production. These above events cause vasodilation. H_2_S inhibits ROS production *via* Nrf-2/HO-1 related redox sensitive signaling pathways. In addition, H_2_S treatment blunts increases in systolic blood pressure by inhibiting inflammation-related signaling pathways. H_2_S, hydrogen sulfide; NO, nitric oxide; Akt, protein kinase B; eNOS, endothelial NO synthase; PKG, protein kinase G; PI3K, phosphoinositide 3-kinase; PPARδ, peroxisome proliferators-activated receptor δ; AMPK, adenosine 5’-monophosphate (AMP)-activated protein kinase; Nrf2, nuclear factor erythroid 2-related factor 2; HO-1, heme oxygenase 1; NF-κB, nuclear factor-kappa B; BMP4, bone morphogenetic protein 4; COX-2, cyclooxygenase-2; NLRP3, nucleotide-binding oligomerization domain, leucine rich repeat, and pyrin domain-containing protein 3.

Importantly, supplementation with S-zofenopril ameliorates vascular endothelial dysfunction by potentiating the H_2_S pathway in spontaneously hypertensive models (Bucci et al., 2014). Moreover, exercise training counteracts hypertension, ameliorates vascular remodeling, and endothelial dysfunction *via* restoring bioavailability of H_2_S and NO in hypertensive rats (Gu et al., 2013). HDAC6 inhibitor tubastatin A alleviates Ang II-induced high blood pressure and vasoconstriction by preventing the protein degradation of CSE (Chi et al., 2019). Overall, these studies suggest that upregulation of H_2_S may be considered as a promising strategy for preventing the progression of hypertension and its associated endothelial dysfunction (**Figure 4**). However, further in-depth research is still required to understand the precise underlying mechanisms, and this will be helpful to develop better therapeutic employment of H_2_S in the treatment of hypertension.

## Evidence for H_2_S/NO Crosstalk in Endothelial Inflammation

Due to the importance of H_2_S and NO in cardiovascular disease, the interactive regulatory functions of H_2_S and NO in endothelial dysfunction-associated cardiovascular disease may be a very attractive subject. In other words, the biological interactions of H_2_S with NO could influence each other’s fate in the endothelium as described previously (Wang et al., 2015b; Nagpure and Bian, 2016; Wu and Hu, 2018) (**Figure 2**).

Studies on H_2_S/NO interaction in inflammation response, especially in endothelial cell inflammation, have been less extensive. Administration of LPS increases H_2_S synthesis, upregulates CSE and iNOS expressions, and promotes myeloperoxidase activity in the liver, whereas the effects are inhibited by the NO donor, nitroflurbiprofen (Anuar et al., 2006). These results suggest that downregulation of H_2_S biosynthesis is responsible for the augmented anti-inflammatory activity of nitroflurbiprofen in the liver (Anuar et al., 2006). In turn, pretreatment with H_2_S is able to inhibit LPS-induced iNOS expression and NO production *via* heme oxygenase 1 (HO-1) expression in macrophages (Oh et al., 2006). In accordance with this finding, H_2_S donor inhibits the release of the pro-inflammatory mediators and NO production, potentially *via* NF-κB inactivation in LPS-treated macrophages (Whiteman et al., 2010). The pulmonary CSE expression and H_2_S levels are downregulated in a model of inflammatory lung disease (Chen et al., 2009; Whiteman and Winyard, 2011). NaHS, a donor for H_2_S, significantly attenuates pulmonary iNOS activation in ovalbumin-treated rats (Chen et al., 2009). Moreover, H_2_S is found to act as an anti-inflammatory agent contributing to gastrointestinal mucosal defense through NO-dependent pathway (Jensen et al., 2017). In cardiovascular system, vasodilation is impaired and endothelial H_2_S content is decreased in vessels from obese mice, this may be attributed to the increased iNOS activity in proinflammatory macrophages (Candela et al., 2017). This finding suggests that macrophages-derived iNOS promotes microvascular endothelial dysfunction through reducing the bioavailability of H_2_S in the blood vessel (Candela et al., 2017). GYY4137, a novel slow-releasing H_2_S compound, is reported to attenuate vascular inflammation and improve endothelial function *via* activating aortic eNOS phosphorylation in ApoE^-/-^ mice (Liu et al., 2013). A number of studies have showed that H_2_S strengthens endothelial NO production *via* activating eNOS phosphorylation (Predmore et al., 2011; Xiao et al., 2018), which results in ameliorating the development of hypertension (Xiao et al., 2018). Taken together, the above studies imply that a complex interaction between H_2_S and NO might serve as an important regulator for endothelial inflammation and associated endothelial dysfunction. However, the potential mechanisms of the interactions between H_2_S and NO in endothelial inflammation remain unclear. As research in this area progresses and more data are available, it will help us to better understand the underlying mechanisms.

## Role of H_2_S in Intestinal Microbiota and Circadian Rhythms

It should be emphasized that intestinal microbiota is also an emerging factor for human health and disease, including cardiovascular diseases (Tang et al., 2017). In recent years, both human and animal experiments have established that alterations in the composition, function, and metabolites of intestinal flora might induce gut microflora dysbiosis, contributing to the pathogenesis of cardiovascular disorders (Tang et al., 2019). Circadian rhythmicity is a characteristic of mammalian metabolism that orchestrates metabolic processes in living organisms based on day/night light cycles (Liu and Chang, 2017). Disturbance of circadian rhythmicity is associated with increased risk for metabolic obesity, diabetes, and cardiovascular dysfunction (Crnko et al., 2018). Similarly, the intestinal microbiota exhibits their own circadian rhythmicity in terms of composition and functions (Tahara et al., 2017). Circadian disorganization may affect the intestinal microbiota which may result in metabolic syndrome and cardiovascular diseases (Voigt et al., 2016). Accumulating evidence has indicated that circadian rhythm disruption in intestinal microbiota is involved in various human diseases, including cardiovascular diseases (Jin et al., 2019). Thereafter, interfering with the composition, function, and metabolites of the intestinal flora or recovery of the normal circadian rhythm in the intestinal flora may provide valuable insights into potential therapeutic strategies for cardiovascular diseases.

Notably, the cysteine degradation by the microbiota is taken as a dominant pathway for H_2_S generation (Basic et al., 2017). Intestinal microbiota is a potential target of H_2_S, and H_2_S acts on gastrointestinal epithelium to modify the gut microbiota (Wallace et al., 2018). It has been reviewed that H_2_S is a double-edge sword for the intestinal epithelium with beneficial effect at low concentration (nanomolar to low micromolar), but deleterious effects at higher concentrations (high micromolar to millimolar) (Blachier et al., 2019). Considering the critical importance of intestinal microbiota and H_2_S in maintaining cardiovascular homeostasis, it is believed that intestinal microbiota-derived H_2_S integrates microbial and circadian cues for regulation of diurnal metabolic rhythms, thereby influencing the endothelial dysfunction in cardiovascular system. However, it is still largely unknown with respect to the roles of H_2_S in intestinal microbiota-mediated endothelial dysfunction. The relationship between H_2_S and intestinal microbiota in cardiovascular regulation may be a very interesting topic. As the gut microbiota leads to much more H_2_S production from cysteine than endogenous metabolism, it is likely that H_2_S from the bacterial or intestinal epithelium may be a critical determinant for cardiovascular health or disease. However, additional investigation is warranted to identify the exact roles of H_2_S in intestinal microbiota and circadian rhythms. Our current understanding of the relationship between H_2_S and intestinal microbiota in endothelial inflammation-related cardiovascular disorders is expanding continuously. The interaction between their signaling pathways is increasingly recognized as the future direction for the research in the gasotransmitters field.

## Concluding Remarks and Future Perspectives

In addition to NO deficiency and intestinal flora, other factors including oxidative stress (Nakahira et al., 2011), endoplasmic reticulum stress (Battson et al., 2017; Luchetti et al., 2017), mitochondrial dysfunction (Gao et al., 2018), hypoxia (Feng et al., 2017a), homocysteine (Esse and Barroso, 2019), and immune activation (Pan et al., 2017) are also closely related with endothelial inflammation and dysfunction in cardiovascular diseases. With in-depth research, our knowledge on the underlying mechanisms of H_2_S-mediated suppression of endothelial cell inflammation is expanding and it is now apparent that interactions between H_2_S and endothelial inflammation-regulated pathways may be proposed as a promising approach for cardiovascular disease therapy. A better understanding of such interactions will be favorable to develop novel therapeutic strategies for endothelial dysfunction-related cardiovascular diseases.

Due to a myriad of biological functions of H_2_S, there has been a growing interest regarding the enormously therapeutic potential of H_2_S in various diseases including cardiovascular diseases. However, our current knowledge on cardiovascular protective effects of H_2_S is mainly from animal or cell experiments using H_2_S donors or inhibitors of H_2_S-producing enzymes. Whether the promising effects of these chemicals in animal studies can be transferable to clinical studies warrants further studies. As such, it should be mentioned that clinical trial results will also pave the way to a better understanding of the effectiveness of H_2_S in human diseases. In one completed clinical trial in healthy volunteers and subjects with impaired renal function received known concentrations of sodium sulfide (clinicaltrials.gov, NCT00879645). Despite that only some results have been announced so far, but the treatment could be considered safe because no serious adverse effects are occurred in the involved patients. However, some caution can be warranted as another clinical trial regarding the potential of H_2_S in coronary artery bypass graft patients was terminated without undisclosed reasons (NCT00858936) and one had been withdrawn before enrollment (NCT01007461). Some other completed trials are completed to test the role of H_2_S in inflammatory diseases such as ulcerative colitis (NCT01282905) or septic shock and stroke (NCT01088490). However, to date, no results are posted due to unknown reasons. Therefore, further results and information from those ongoing and future trials will help to elucidate the physiological and pathophysiological importance of H_2_S in various diseases.

Until now, endothelial inflammation and dysfunction remain mortal factors for cardiovascular diseases. It is anticipated that a full understanding of the modulatory mechanisms of the link between endothelial inflammation and destructive H_2_S bioavailability might promote the translation of H_2_S biology to clinical management of endothelial dysfunction-related cardiovascular diseases. To achieve this, more original work remains to be experimentally evaluated in the future.

## Author Contributions

H-JS and J-SB designed the contents of this review article. H-JS, Z-YW, and X-WN conducted initial search of literature and prepared the figures. H-JS and J-SB drafted the manuscript. J-SB critically helped to revise the manuscript. All authors have read and approved the final manuscript.

## Funding

This work was supported by the Ministry of Education of Singapore Tier 2 Research grant (MOE2017-T2-2-029), and the China Jiangsu Nature Science Foundation (BK20181185).

## Conflict of Interest

The authors declare that the research was conducted in the absence of any commercial or financial relationships that could be construed as a potential conflict of interest.

## Abbreviations

3-MP, 3-mercaptopyruvate; 3-MST, 3-mercaptopyruvate sulfurtransferase; ACE2, angiotensin converting enzyme 2; AMPK, adenosine 5’-monophosphate (AMP)-activated protein kinase; Ang-(1-7), angiotensin 1-7; BKCa, voltage- and calcium-gated potassium; BMP4, bone morphogenetic protein 4; CAT, cysteine aminotransferase; CBS, cystathionine β-synthase; CO, carbon monoxide; COX-2, cyclooxygenase-2; CSE, cystathionine γ-lyase; EDRF, endothelium-derived relaxing factor; H_2_S, hydrogen sulfide; HDAC6, histone deacetylase 6; HO-1, heme oxygenase 1; ICAM-1, intercellular adhesionmolecule-1; MAPK, mitogen activated protein kinase; Keap1, kelch-like ECH-associated protein 1; L-NAME, Nw-nitro-L-argininemethyl ester; MCP-1, monocyte chemoattractant protein-1; mTOR, mammalian target of rapamycin; NaHS, sodium hydrosulfide; NF-κB, nuclear transcription factor-κB; NO, nitric oxide; Nrf2, nuclear factor erythroid 2-related factor 2; ox-LDL, oxidized low density lipoprotein; PKC, protein kinase C; RAS, renin angiotensin system; ROS, reactive oxygen species; SHR, spontaneously hypertensive rats; STAT3, signal transducer and activator of transcription 3; TNF-α, tumour necrosis factor-α; VCAM-1, vascular cell adhesionmolecule-1; VSMCs, vascular smooth muscle cells; VEGF, vascular endothelial growth factor; VEGFR2, vascular endothelial growth factor receptor 2.
